# Evaluation of desmin as a diagnostic and prognostic marker of childhood rhabdomyosarcomas and embryonal sarcomas.

**DOI:** 10.1038/bjc.1987.203

**Published:** 1987-09

**Authors:** P. Dias, P. Kumar, H. B. Marsden, P. H. Morris-Jones, J. Birch, R. Swindell, S. Kumar

**Affiliations:** Christie Hospital and Holt Radium Institute, Manchester, UK.

## Abstract

**Images:**


Br. J. Cancer (1987) 56, 361–365

© The Macmillan Press Ltd., 1987

### Evaluation of desmin as a diagnostic and prognostic marker of childhood rhabdomyosarcomas and embryonal sarcomas

P. Dias, P. Kumar, H.B. Marsden, P.H. Morris-Jones, J. Birch, R. Swindell & S. Kumar

*Christie Hospital and Holt Radium Institute, Manchester M20 9BX, UK.*

**Summary** The diagnostic and prognostic relevance of desmin expression in 80 rhabdomyosarcomas (RMS) and 5 embryonal sarcomas (ES) was examined using a peroxidase anti-peroxidase staining procedure. Fifty-nine RMS but only one ES stained for desmin ($P<0.05$). The maximum percentage of desmin containing cells was 49 in RMS compared with only 1% in ES. Desmin positivity correlated inversely with survival ($P<0.02$) in that RMS with high proportions of desmin positive cells were associated with poorer prognoses than those containing fewer desmin positive cells. If the degree of expression of desmin is related to myogenic differentiation, then our results indicate that poorly differentiated RMS tend to have a better prognosis than the well differentiated tumours. One possible explanation is that the poorly differentiated RMS respond better to chemotherapy than to well differentiated RMS. A multivariant analysis incorporating desmin staining, treatment, histology, age and gender revealed that the two most significant independent prognostic factors were treatment and histology.

Childhood rhabdomyosarcomas (RMS) are a heterogenous group of tumours of skeletal muscle elements varying from poorly differentiated to well differentiated forms. The difficulty in diagnosis, especially of poorly differentiated forms is well documented, and is often due to a lack of observable features of skeletal muscle differentiation in the tumour cells. Recently a number of skeletal muscle markers have been introduced which aid the diagnosis of RMS (For review see Roholl *et al.*, 1986). Generally the expression of most skeletal muscle markers depends on the degree of differentiation of tumour cells. For instance, although skeletal muscle myosin and myoglobin are highly specific markers for the detection of skeletal muscle cell differentiation in RMS, the use of these markers is limited to well differentiated tumours. The alternative use of the muscle isoenzyme of creatine kinase, which is expressed in a higher proportion of poorly differentiated rhabdomyosarcomas (de Jong *et al.*, 1985 and personal communication), is somewhat limited in that this isoenzyme is also expressed in leiomyosarcomas, malignant fibrous histiocytomas, ganglioneuroblastomas, fibrosarcomas and liposarcomas (Wold *et al.*, 1981; de Jong *et al.*, 1985; Roholl *et al.*, 1986).

Although the presence of desmin in tumours has been shown to vary from 32% to 100% in different studies (see Table I), it is expressed in RMS of a wide spectrum of differentiation and has consequently been advocated as a reliable marker for distinguishing poorly differentiated RMS from other small round cell tumours in childhood (Altmannsberger *et al.*, 1985; Harms *et al.*, 1985; de Jong *et al.*, personal communication; Tsokos *et al.*, personal communication). Most of these studies however, were limited in that they were carried out on relatively small numbers of tumours. In addition none of these reports has actually evaluated the prognostic relevance of desmin in RMS. In the present study the reliability of desmin as a diagnostic and prognostic marker has been examined in a large number of childhood RMS.

### Materials and methods

### *Tissues*

Formalin fixed, paraffin embedded tumour specimens of 80 RMS and 5 embryonal sarcomas were stained with desmin antibody (Table II). Haematoxylin and eosin (H&E) stained sections of these tumours were examined and classified according to the system of the International Society of Paediatric Oncology (SIOP) (Marsden 1985; & unpublished data) (Table II).

The embryonal sarcomas included in this study were histologically similar to RMS, although on routine staining they showed no evidence of myoblastic differentiation such as eccentric nuclei and eosinophilic cytoplasm.

### *Antisera*

The rabbit polyclonal antibody to desmin (Euro Diagnostics, Holland) was raised against chicken gizzard, affinity purified and its specificity was established by immunoblotting and by extensive testing using both frozen and paraffin embedded tissues (Altmannsberger *et al.*, 1985). It was diluted 1:25 for staining. A monoclonal antibody to desmin was purchased from Amersham International plc. UK and used at a dilution of 1:10.

### *Staining procedures*

A peroxidase anti-peroxidase (PAP) staining procedure was carried out following the details given by Polak and Van Noorden (1983). Sections were deparaffinized with xylene, rehydrated in graded ethanols, washed with 0.1 M phosphate buffered saline (PBS), pH 7.4, followed by thorough rinsing with tap water. Endogenous peroxidase activity was inhibited by treating sections with 0.5% hydrogen peroxide in methanol. The sections were washed and incubated with a 1/20 dilution of normal swine serum (Sera-Labs, UK) for 15 min. The excess normal blocking serum was drained off and the sections incubated with desmin polyclonal antiserum for 1 h, after which the sections were washed in 3 changes of PBS (5 min each) and treated with a 1/50 dilution of swine anti-rabbit immunoglobulin (Dakopatts, Mercia Brocades, UK) for 30 min. After washing in 3 changes of PBS, the sections were incubated with a 1/40 dilution of rabbit peroxidase anti-peroxidase complex (Dakopatts) for 30 min and finally washed in 3 changes of PBS. The colour was developed using freshly prepared diaminobenzidine (DAB, Sigma) as the chromogen. After thorough washing, the sections were counterstained with haematoxylin, washed, dehydrated and mounted.

For staining with desmin monoclonal antibody the indirect immunoperoxidase method was used. Essentially the procedure was same as the PAP procedure except the reagents substituted were a 1/20 dilution of normal rabbit serum, followed by a 1/10 dilution of desmin monoclonal antibody and finally by a 1/40 dilution of peroxidase conjugated rabbit anti-mouse immunoglobulin (Dakopatts).

Correspondence: S. Kumar.
Received 13 April 1987; and in revised form, 4 June 1987.

**Table I** The results of desmin staining in rhabdomyosarcomas reported in the literature

| *Reference* | *Nos of desmin positive tumours: Total examined* | *Per cent desmin positive tumours* |
|---|---|---|
| Kahn *et al.* (1983) | 8/25 | 32 |
| Molenaar *et al.* (1985) | 17/21 | 81 |
| Mordechai *et al.* (pers. comm.) | 14/15 | 93 |
| De Jong *et al.* (pers. somm.) | 33/38 | 87 |
| Tsokos *et al.* (pers. comm.) | 11/11 | 100 |
| Altmannsberger *et al.* (1985) | 25/25 | 100 |
| Present study | 59/80 | 74 |

Staining of tumours was recorded as positive or negative. Irrespective of the intensity of staining, the proportion of positive cells was ascertained for each desmin positive tumour by examining 10 randomly selected fields at a magnification of ×700 using a Leitz microscope fitted with an eye piece graticule. Between 500 and 700 cells were scored as positive or negative for each slide and the results expressed as the percentage of desmin-positive cells per tumour.

The standard Kaplan and Meier statistical analysis (Kaplan & Meier, 1958) was used for calculating and projecting curves of overall survival. Statistical differences between the survival curves were analysed by the log rank test (Peto *et al.*, 1977). These data were further analysed using a multivariant analysis (Cox *et al.*, 1972).

### Results

None of the negative control tissue sections showed any staining with normal rabbit serum (used alone without anti-desmin antibody). For each tumour, identical staining results were obtained using polyclonal and monoclonal antibodies although the staining intensity was much stronger with the former. For this reason, the percentages of desmin positive tumour cells were scored on sections stained with polyclonal antibody.

Fifty-nine of 80 (74%) of RMS were positive for desmin compared with 1 of 5 ES ($P<0.05$, Fisher's exact test; Table III & Figure 1 A–F). The percentage of desmin positive cells per tumour varied from 0–49%. The mean percentage of desmin positive cells for each sub-group ranged from 0.2 to 16.7% (Table IV). The intensity of the staining reaction varied not only from tumour to tumour but also among neoplastic cells in a given tumour in which both weakly and strongly positive foci of cells were seen. Generally, a higher proportion of well-differentiated tumour cells, such as strap cells and multinucleated giant cells stained for desmin although staining was also observed in some small, apparently poorly differentiated tumour cells (Figure 1 A, B).

**Table II** Rhabdomyosarcomas and embryonal sarcomas (classified as per International Society of Paediatric Oncology) used to stain with desmin antibody

| *Histological classification* | *No. examined* |
|---|---|
| A. Rhabdomyosarcomas | |
| (i) Loose (botryoid and non-botryoid) | 15 |
| (ii) Dense (poor and good myoblastic differentiation) | 43 |
| (ii) Alveolar | 19 |
| (iv) Pleomorphic (adult type) | 3 |
| B. Embryonal sarcoma | 5 |
| TOTAL | 85 |

**Table III** Results of desmin staining of 80 rhabdomyosarcomas and 5 embryonal sarcomas

| | *No. positive / No. examined* | *Per cent desmin positive* |
|---|---|---|
| Histology | | |
| A. Rhabdomyosarcomas | | |
| (i) Loose | 14/15 | 93 |
| (ii) Dense | 28/43 | 65 |
| (iii) Alveolar | 14/19 | 74 |
| (iv) Pleomorphic (adult type) | 3/3 | 100 |
| | 59/80 | 74 |
| B. Embryonal sarcomas | 1/5 | 20 |

**Table IV** The results of desmin staining expressed as percentage of desmin positive cells in rhabdomyosarcomas and embryonal sarcomas

| *Histology* | *Percent positive cells (Mean+s.d.)* | *Median* | *(range)* |
|---|---|---|---|
| Loose | 10.78+13.24 | 5.5 | (0–49) |
| Dense | 9.0 + 9.4 | 4 | (0–32) |
| Alveolar | 11.78+11.93 | 8 | (0–36) |
| Pleomorphic (adult type) | 16.66+ 7.7 | 17 | (7–32) |
| Embryonal sarcoma | 0.2 + 0.4 | 1 | (0– 1) |

### *Staining and prognosis*

Figure 2A shows that although there was a trend towards negatively stained tumours having a better prognosis than positively stained tumours, the difference between the two groups was not statistically significant. However, when the tumours were graded according to the percentage of desmin positive cells into four groups (0%, 1–4%, 5–19% and 20+%), a statistically significant difference in prognosis was observed among them ($P<0.02$). The negatively stained group (0%) and the group with only 1–4% desmin positive cells had a better prognosis than those with higher percentages (>5%) of desmin positive cells (Figure 2B).

There appeared to be a difference in prognosis between the subgroups as regards histological classification (Figure 3) although this was not statistically significant ($P<0.06$). However, histology assumed significance ($P<0.03$) once those patients receiving no chemotherapy (i.e. those diagnosed prior to 1970) were excluded from the analysis. In the present somewhat small series, gender and site had no effect on prognosis (Figure 3).

A multivariant analysis incorporating desmin staining, histology, age, sex, primary site of occurrence and chemotherapy as prognostic factors revealed that the two most important independent prognostic factors were histological classification and treatment.

**Figure 1** Immunoperoxidase staining of formalin fixed, paraffin embedded rhabdomyosarcomas (RMS) cells with desmin antibody. a and b. Show staining with desmin antibody of an undifferentiated small round cell tumour. (**a**). Stained with the desmin antibody. (**b**). H&E of the same tumour. (**c**). Strong staining of a well differentiated dense RMS. (**d**). Another well differentiated RMS that stained intensely with the desmin antibody. It should be noted that this magnification was used for assessing the percentage of desmin positive and negative cells. (**e**). Botryoid RMS containing many desmin positive cells. An epithelial lining can be seen in the top left hand corner. (**f**). Botryoid RMS containing only a small percetage of desmin positive cells.

### Discussion

The aim of this study was to assess the reliability of desmin as a diagnostic marker of rhabdomyosarcoma and to evaluate its prognostic relevance. The staining result showed that 74% (59 out of 80) rhabdomyosarcomas were positive for desmin. When five embryonal sarcomas were included, 71% (60 of 85) of the tumours were positive for desmin. Our desmin staining results are within the range reported in the literature where the expression of desmin has been shown to vary from 32 to 100 percent (see Table I). The variation in positivity may be partly accounted for by the use of different antibodies, the degree of differentiation of the tumours examined (de Jong *et al.* 1985; Tsokos *et al.*, personal communication; Molenaar *et al.*, 1985) and the method of fixation and staining of the tumour specimens (Altmannsberger *et al.*, 1985; Molenaar *et al.*, 1985). It appears that formalin fixation may not be conducive to immunostaining for desmin, especially in poorly differentiated rhabdomyosarcomas. In their comparison of frozen with formalin fixed RMS, Molenaar *et al.* (1985) concluded that the latter is inadequate for demonstrating minimal amounts of desmin. In the desmin positive tumours in our series, a higher proportion of well differentiated tumour cells stained for desmin whereas most of the poorly differentiated tumour cells were negative. It is likely that some of these negatively stained cells may have expressed minimal amounts of desmin which were not detected after formalin fixation.

**Figure 2** Life Table: Survival in years. (**a**). Desmin staining and survival in RMS: (a) desmin negative (b) desmin positive. (**b**). Percent desmin staining cells and survival: RMS with (a) 0% desmin positive cells (b) 1–4% desmin positive cells and (c) >5% desmin positive cells.

**Figure 3** Life Tables: Survival in years. (**a**). Histology (SIOP classifications) and survival: (a) loose RMS (b) embryonal sarcoma (c) alveolar RMS (d) dense RMS and (e) pleomorphic RMS. (**b**). Age and survival (a) >3 year (b) <1 year and (c) 1–3 years. (**c**). Gender and survival: (a) male and (b) female. (**d**). Site and survival: (a) head and neck (b) trunk (c) extremities.

This might also have been the case with the totally desmin negative tumours.

The mean percentages of desmin positive cells in the subgroups of RMS varied from 9.0 to 16.7 percent. The highest percentage (16.7%) was found in the pleomorphic, and the lowest (9.0) in dense RMS, however these differences were not significant. Since there are no reports on the actual quantitation of desmin containing cells in RMS, it is not possible to compare our results with the published literature. It is noteworthy that only one of the 5 ES was desmin positive and the percentage of positive cells was low (1.0%). Thus, the rarity of desmin positive cells in ES agrees with the histological classification i.e. lends support to the separation of ES from RMS.

There was a trend towards desmin negative tumours having a better prognosis, although the difference between the survival curves was not statistically significant. However, it was found that the two groups with 0% and 1–4% desmin positive cells had a better prognosis than those with a higher percentage of cells staining (5+%). Thus the results of this study appear to indicate that there is an inverse correlation between the number of cells expressing desmin in RMS and prognosis. The multivariant analysis established that this was not an independent prognostic factor, whereas histological classification and chemotherapy were.

Some studies support the association of histology with prognosis whereas others maintain that histology is not a prognostic factor (for review see Triche, 1982 and Favara *et al.*, 1986). The published literature indicates that desmin-positivity in RMS is associated with cytological differentiation. Molenaar *et al.* (1985) noted that the differences in staining observed between their poorly, moderately and well differentiated RMS reflected quantitative differences in the expression of desmin. Therefore, if desmin is utilised as a marker of myogenic differentiation, the results of this study would indicate that poorly differentiated rhabdomyosarcomas tend to have a better prognosis than well differentiated tumours. Molenaar *et al.* (1984) concluded that the major role of chemotherapy in RMS was the selective destruction of undifferentiated tumour cells and similar findings have been reported by Harms *et al.* (1985). Therefore, the better prognosis of desmin negative RMS noted by us might be due to the fact that poorly differentiated rhabdomyosarcomas respond better to chemotherapy with the majority of their (undifferentiated) cells being selectively killed probably because they are in continuous cell cycle. The more differentiated tumours may be relatively resistant to chemotherapy in that most of their cells would be cycling more slowly and would not be killed. Further studies involving the use of other markers for RMS differentiation and proliferation need to be undertaken to extablish the validity of this hypothesis. It is interesting that an inverse relationship between differentiation and survival has also been noted in medullablastoma. Thus, Latchaw *et al.* (1985) reported that tumours showing no differentiation had four year survival rate of 70% compared with 32% for those showing differentiation.

Technical assistance of Mrs Joan Ashworth is most gratefully acknowledged.

### References

ALTMANNSBERGER, M., WEBER, K., DROSTE, R. & OSBORN, M. (1985). Desmin is a specific marker for rhabdomyosarcomas of human and rat origin. *Am. J. Pathol.*, **118**, 85.

COX, D.R. (1972). Regression models and life tables (with discussion). *J.R. Statist. Soc., B*, **34**, 187.

FAVARA, B.E., GALLIANI, C.A. & WAKLEY, P.E. (1986). Advances in the care of the child with cancer. The importance of histologic subclassification of tumours. *Cancer* (Suppl.), **58**, 426.

HARMS, D., SCHMIDT, D. & TREUNER, J. (1985). Soft tissue sarcomas in Childhood. A study of 262 cases including 169 cases of rhabdomyosarcomas. *Z. Kinderchir.*, **40**, 140.

DE JONG, A.S.H., VAN KESSEL-VAN VARK, V., ALBUS-LUTTER, C.H.E. & VOUTE, P.A. (1985). Creatine kinase subunits M and B as markers in the diagnosis of poorly differentiated rhabdomyosarcoma in children. *Human Pathol.*, **16**, 924.

KAHN, H.J., YEGER, H., KASSIM, O. & 5 others (1983). Immunohistochemical and electron microscopic assessment of childhood rhabdomyosarcoma. *Cancer*, **55**, 1897.

KAPLAN, E.L. & MEIER, P.J. (1958). Non-parametric estimation from incomplete observations. *J. Amer. Statist. Ass.*, **53**, 457.

LATCHAW, J.P., HAHN, J.F., MOYLAND, D.J., HUMPHRIES, R. & MEALEY, J. (1985). Medulloblastoma: Period of risk reviews. *Cancer*, **55**, 186.

MARSDEN, H.B. (1985). The pathology of soft-tissue sarcomas with emphasis on childhood tumours. In *Bone tumours and soft-tissue sarcomas*, D'Angio, G.J. & Evans, A.E. (eds) p. 14. Edward Arnold: London.

MOLENAAR, W.M., OOSTERHUIS, J.W. & KAMPS, W.A. (1984). Cytologic differentiation in childhood rhabdomyosarcomas following polychemotherapy. *Human Pathol.*, **15**, 973.

MOLENAAR, W.M., OOSTERHUIS, J.W., OOSTERHUIS, A.M. & RAMAEKERS, F.C.S. (1985). Mesenchymal and muscle-specific intermediate filaments (Vimentin and desmin) in relation to differentiation in childhhod rhabdomyosarcoma. *Human Pathol.*, **16**, 838.

PETO, R., PIKE, M.C., ARMITAGE, P. & 7 others (1977). Design and analysis of randomised clinical trials requiring prolonged observations of cALL patients. II. Analysis and examples. *Br. J. Cancer*, **35**, 1.

POLAK, J.M. & VAN NOORDEN, S.V. (eds) (1983). Immunocytochemistry: Practical applications in pathology and biology. p. 11. Wright P.S.G.: Bristol

ROHOLL, P.J.M., DE JONG, A.S.H. & RAMAEKERS, F.C.S. (1986). Diagnostic markers in soft tissue tumous. In *Management of soft tissue and bone sarcomas*, van Oosterom, A.T. & van Unnik, J.A.M. (eds) p. 35. Raven Press: New York.

TRICHE, T. (1982). Pathology of cancer in the young. In *Cancer in the Young*, Levine, A.S. (ed) p. 119. Year Book Medical Publishers: Chicago.

WOLD, L.E., LI, C.Y., HOMBURGER, H.A. (1981). Localization of the B and M polypeptide subunits of creatine kinase in normal and neoplastic tissues by an immunoperoxidase technic. *Am. J. Clin. Pathol.*, **75**, 327.


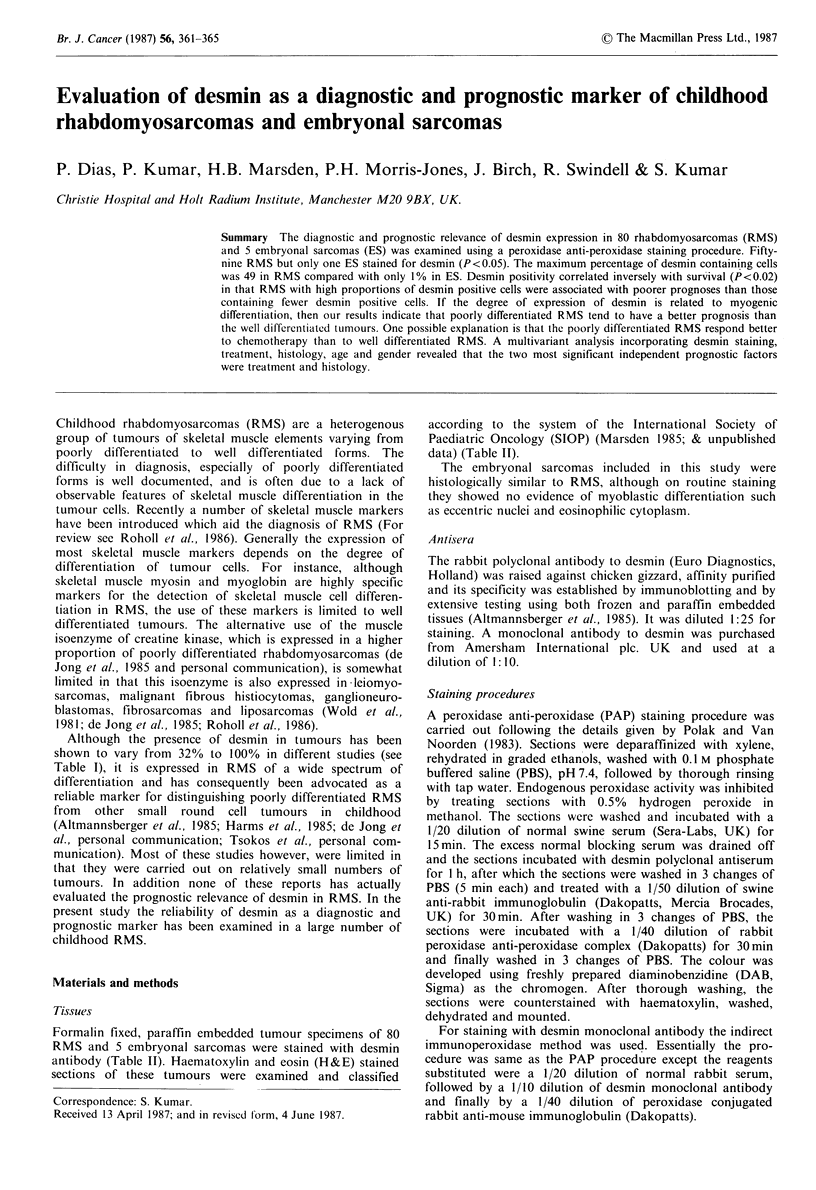

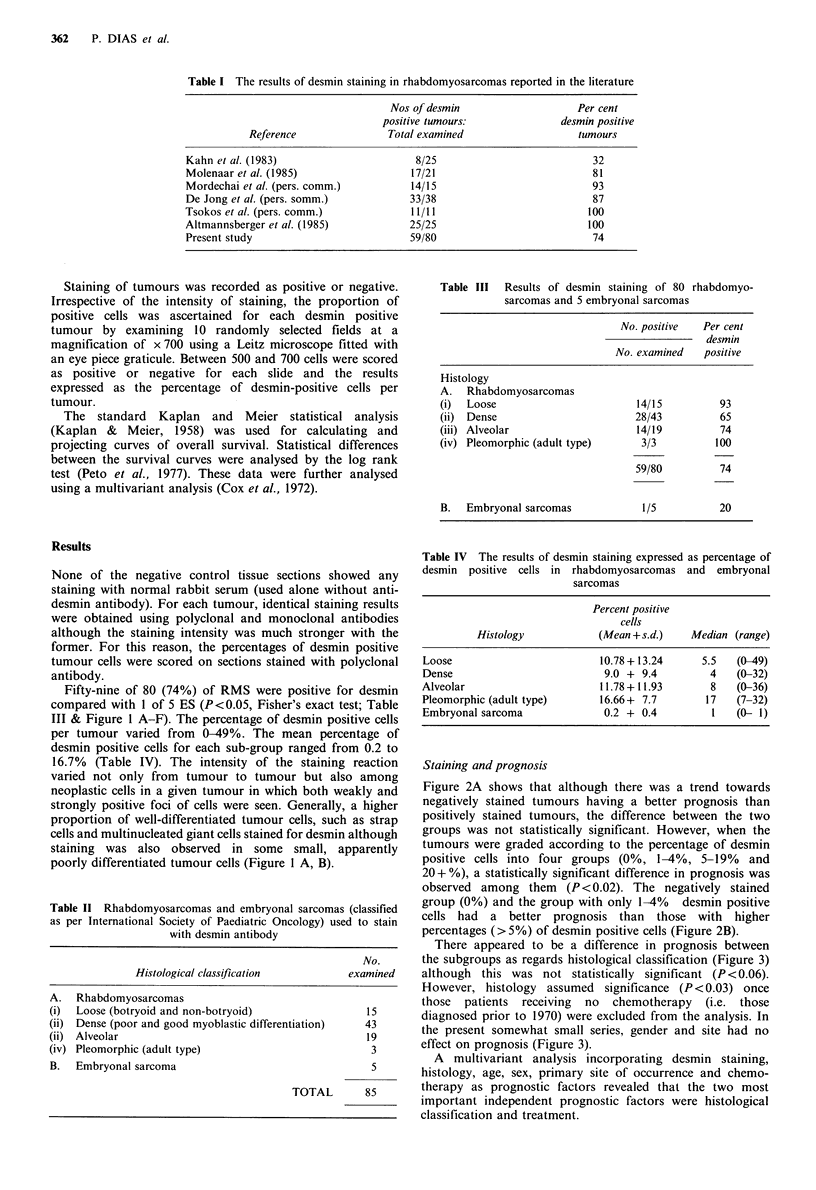

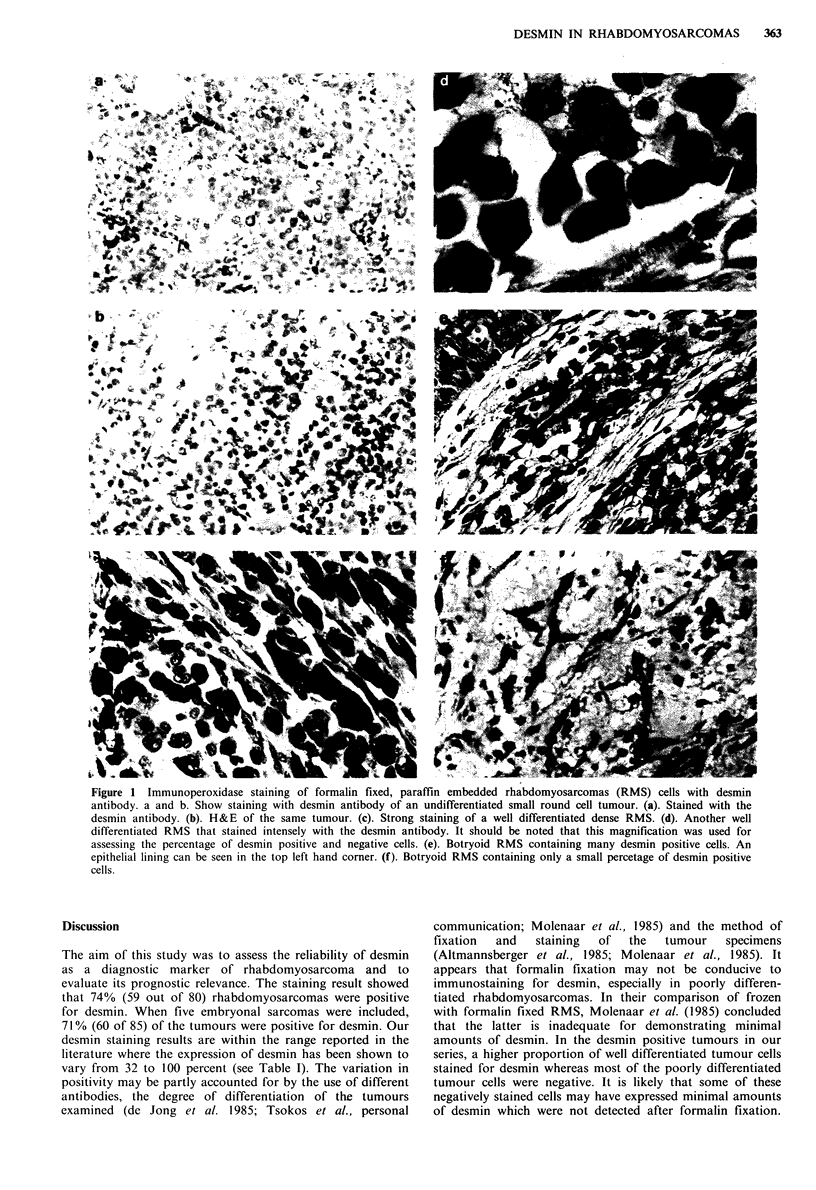

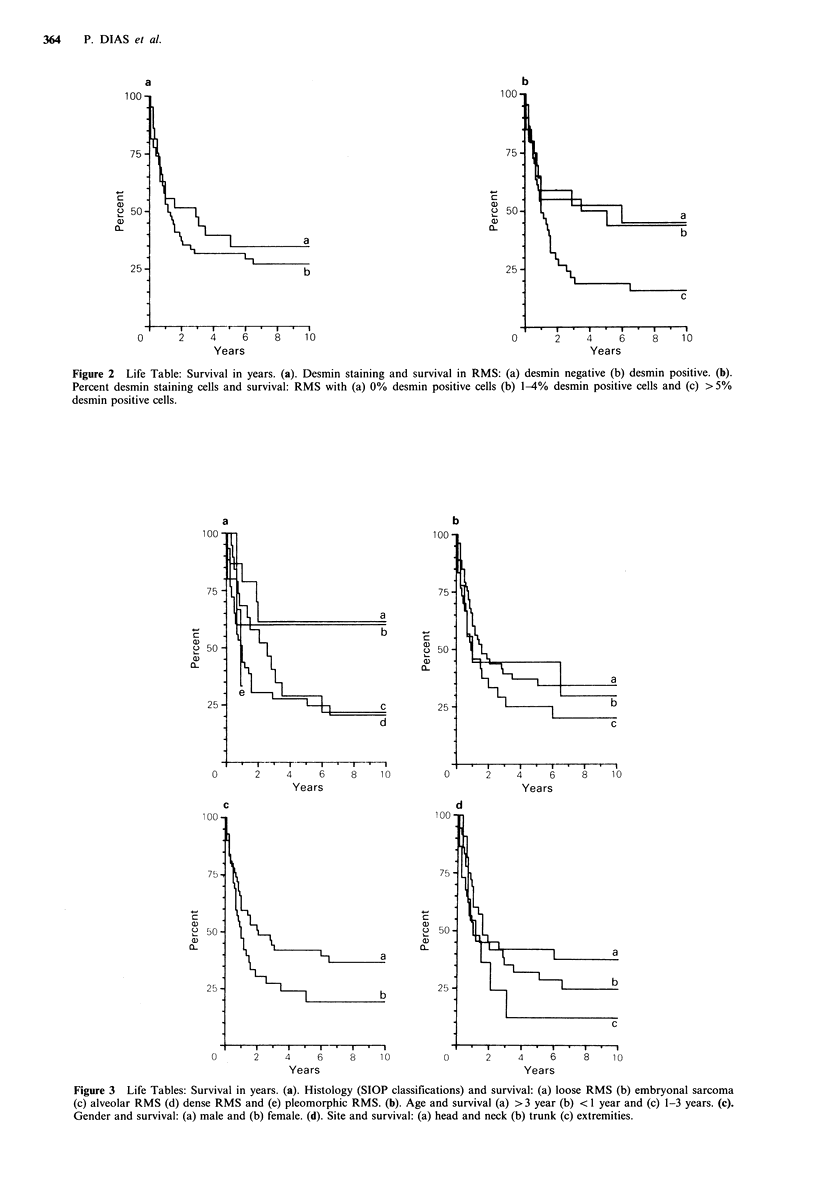

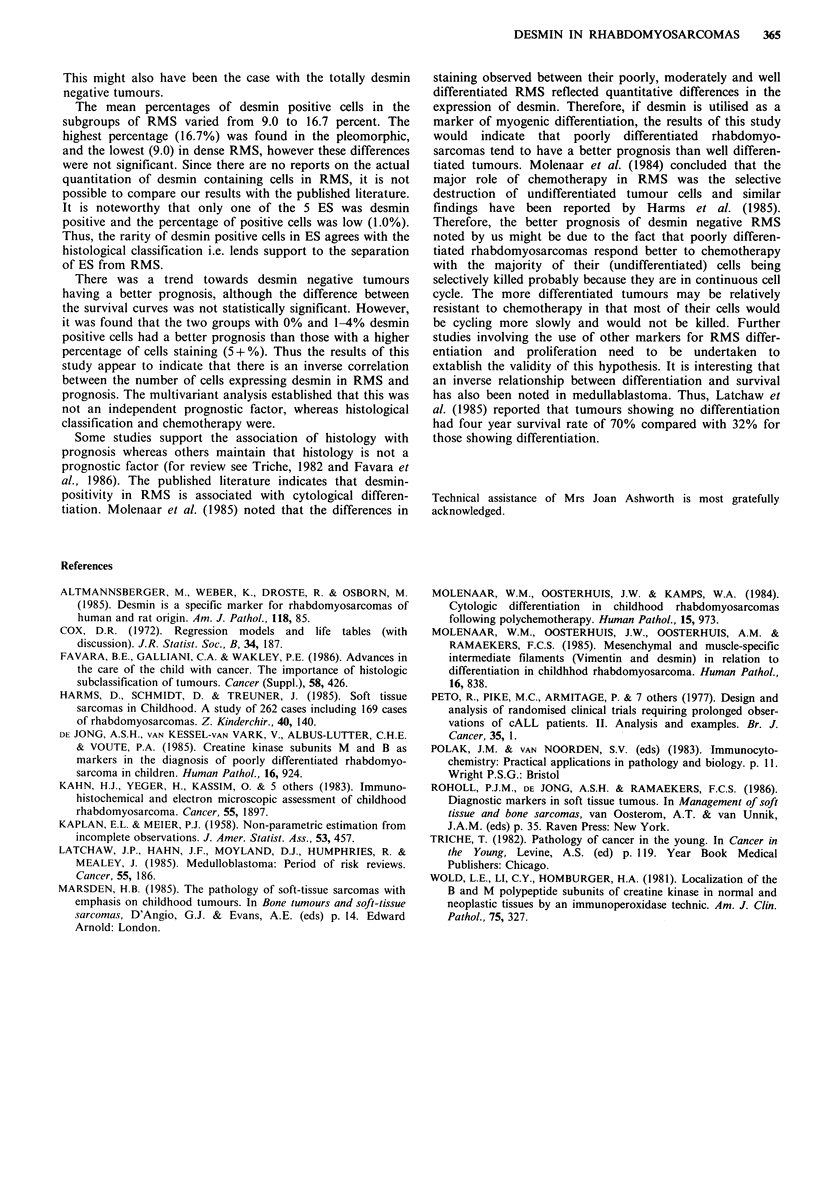


## References

[OCR_00513] Altmannsberger M., Weber K., Droste R., Osborn M. (1985). Desmin is a specific marker for rhabdomyosarcomas of human and rat origin.. Am J Pathol.

[OCR_00522] Favara B. E., Galliani C. A., Wakely P. E. (1986). Advances in the care of the child with cancer. The importance of histologic subclassification of tumors.. Cancer.

[OCR_00527] Harms D., Schmidt D., Treuner J. (1985). Soft-tissue sarcomas in childhood. A study of 262 cases including 169 cases of rhabdomyosarcoma.. Z Kinderchir.

[OCR_00538] Kahn H. J., Yeger H., Kassim O., Jorgensen A. O., MacLennan D. H., Baumal R., Smith C. R., Phillips M. J. (1983). Immunohistochemical and electron microscopic assessment of childhood rhabdomyosarcoma. Increased frequency of diagnosis over routine histologic methods.. Cancer.

[OCR_00547] Latchaw J. P., Hahn J. F., Moylan D. J., Humphries R., Mealey J. (1985). Medulloblastoma. Period of risk reviewed.. Cancer.

[OCR_00558] Molenaar W. M., Oosterhuis J. W., Kamps W. A. (1984). Cytologic "differentiation" in childhood rhabdomyosarcomas following polychemotherapy.. Hum Pathol.

[OCR_00563] Molenaar W. M., Oosterhuis J. W., Oosterhuis A. M., Ramaekers F. C. (1985). Mesenchymal and muscle-specific intermediate filaments (vimentin and desmin) in relation to differentiation in childhood rhabdomyosarcomas.. Hum Pathol.

[OCR_00592] Wold L. E., Li C. Y., Homburger H. A. (1981). Localization of the B and M polypeptide subunits of creatine kinase in normal and neoplastic human tissues by an immunoperoxidase technic.. Am J Clin Pathol.

[OCR_00532] de Jong A. S., van Kessel-van Vark M., Albus-Lutter C. E., Voûte P. A. (1985). Creatine kinase subunits M and B as markers in the diagnosis of poorly differentiated rhabdomyosarcomas in children.. Hum Pathol.

